# Fable, c. 1600

**DOI:** 10.3201/eid0805.020500

**Published:** 2002-05

**Authors:** Polyxeni Potter

**Figure:**
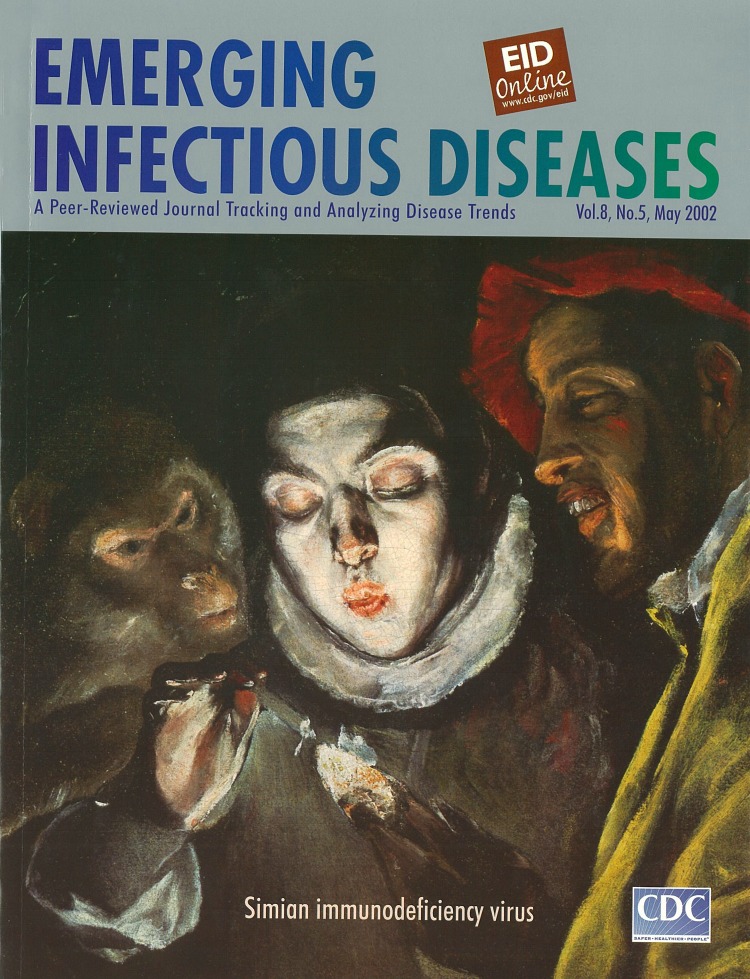
**Fable, c. 1600** (oil on canvas, 49 cm x 54 cm). Domenikos Theodokopoulos (known as El Greco, 1541–1614). Courtesy of The Prado Museum, Madrid, Spain.

**The painting.** El Greco’s Fable is an enigmatic work of art whose meaning and date of origin have provoked much speculation among art historians.

 In the center of the painting, a young boy (or perhaps a girl?) is blowing at a fire held with the left hand, trying to revive the flame to light a small candle held in the right hand. To the left, behind the youth, a chained monkey with an intelligent expression gazes attentively at the fire and also seems to be blowing at it. And to the right, the painting closes with the profile of a man wearing a bright red cap and a mocking grin on his bearded face.

 El Greco painted more than one version of “the blower." An earlier version depicts only a young boy lighting a candle; a later version shows three figures (as seen here).

 During his stay in Rome, El Greco moved in intellectual circles and studied literary sources, among them work by Pliny the Elder, a Roman writer from the First century A.D. Pliny’s writings refer to a painter from antiquity remembered for his painting of a young boy blowing at a fire and for the light reflected from the fire on the boy’s face and in the room. This text may have served as inspiration to El Greco.

 Even though an occult humanistic meaning is possible, interpreting the version of the painting with only one central figure is not too much of a challenge; it could even be a study by the painter of the effects of artificial light on colors. On the other hand, in the version with the three figures, the expressions of the man and the monkey imply that El Greco may have intended to illustrate a fable or allegory, the meaning of which remains elusive. 

**About the painter**. El Greco (meaning “the Greek”) was born in Crete. Details of his early life and training are sketchy, but he probably studied painting in his youth. Although his early work has not survived he probably painted in the late Byzantine style popular in Crete in his time. Reminders of the style are seen in his later works, which are all icons.

Around 1566, El Greco went to Venice, where he studied under Titian and was strongly influenced by Tintoretto, both masters of the High Renaissance. Further Italian inspiration came during the years the artist spent in Rome (1570–1576), where he met several Spaniards associated with the church in Toledo. The Christian doctrines of Spain had a great influence on his approach to painting—his art is filled with passion and restraint, religious fervor and Neoplatonism, as well as the mysticism of Counter-Reformation. 

He arrived in Spain in 1577, possibly attracted by the chance of working on the decorations at Escorial. Although he did not produce any paintings there, his “Martyrdom of St. Maurice” is at the monastery. In the intellectual, passionate, and somewhat pessimistic milieu of Toledo, El Greco created a highly personal pictorial world in the Mannerist style, for which he was acclaimed as on the of the most original artists in his adopted country. 

El Greco’s later paintings (of which Fable is an example) exude a cultivated spirituality and feverish intensity and seem to pulsate with an eerie light generated by the figures themselves. He moved toward unusual colors, groupings, and figure proportions. The figures became increasingly elongated and the canvases manifest “horror vacui,” (dread of unfilled spaces). Subjects of classical mythology attest to El Greco’s humanistic learning and his brilliantly personal and novel approach to traditional themes. The artist died in Toledo in 1614 and was buried there in Santo Domingo el Antiguo.

Sources: The Prado Museum, Madrid, Spainhttp://sunsite.dk/cgfa/greco/greco_bio.htm http://www.artchive.com/artchive/E/el_greco.html

